# The anisotropy of personal space

**DOI:** 10.1371/journal.pone.0217587

**Published:** 2019-06-04

**Authors:** Robin Welsch, Christoph von Castell, Heiko Hecht

**Affiliations:** Department of Psychology, Johannes Gutenberg-Universität Mainz, Mainz, Germany; Rice University, UNITED STATES

## Abstract

Violations of personal space are associated with discomfort. However, the exact function linking the magnitude of discomfort to interpersonal distance has not yet been specified. In this study, we explore whether interpersonal distance and discomfort are isotropic with respect to uncomfortably far or close distances. We also extend previous findings with regard to intrusions into personal space as well as maintenance of distances outside of personal space. We presented subjects with 15 interpersonal distances ranging from 40 to 250 cm and obtained verbal and joystick-based ratings of discomfort. Whereas discomfort rose immediately when personal space was entered, the gradient was less steep for distances that exceeded the comfort region of personal space. Thus, personal space is anisotropic with regard to experienced discomfort.

## Introduction

As a stranger approaches us, there comes a point where we start to feel uncomfortable and intruded upon. Our feeling of an inappropriately large or short distance with respect to another person can be conceived of as a personal space (PS) requirement accompanied by a feeling of psychological distance. How PS extends and shapes distance-behavior has first been studied in animals. For example, animals in captivity claim a relatively smaller territory and flight zone, as compared to wild animals [1]. Sommer [2] pioneered proxemic research in humans. He observed that when interacting with others in a hospital, schizophrenic patients claimed a larger portion of space to themselves as compared to non-schizophrenic patients. Hall [3] took up the idea of interaction distances and proposed four distinct spaces by their radius, mainly based on the appropriateness of potentially available sensory perceptions: intimate space (0–45 cm), personal space (45–120 cm), social space (120–365 cm), and public space (365–762 cm). These ranges have been replicated within a large set of different nationalities and cultures [4], in various measures of interpersonal distance (IPD, [5]) as well as in virtual environments [6–9].

The most prominent definition of PS stems from Leslie Hayduk [10]: „… we can define personal space as the area individual humans actively maintain around themselves into which others cannot intrude without arousing discomfort.“(p.118). This definition structured proxemic research and improved the conceptualization, the measurement of PS, and the identification of correlates.

### Measuring and conceptualizing PS

Attempts to refine the concept of PS have confronted the issue of measuring the shape of PS. For example, Hecht et al. [7] let participants approach both a real and a virtual confederate from multiple angles while measuring the preferred IPD. They could show that PS is approximately circular, both in real and virtual environments. Thus, in line with the definition of PS [10], personal space forms a circular area surrounding the person.

A large set of personal and contextual determinants of the size of PS could be identified [5], mainly due to the development of the stop-distance paradigm. Williams [11], a student of Sommer, let a confederate walk up to a subject until the subject deemed the distance to be most comfortable for conversation, and signaled the confederate to stop. The resulting IPD was measured as an approximation of the size of PS. This approach to PS has since been adopted in proxemic research, with some minor variations, which include subjects actively approaching the experimenter or a confederate [12], or projective techniques such as chair placement [13].

Although the stop-distance procedure appears to be the most reliable and valid approach [5], studies differ greatly in the average size of PS. Some studies found strangely short preferred IPDs at about 35 cm, [14] or quite large IPDs with more than 120 cm [9, 15, 16]. This is problematic in many ways. Firstly, it is particularly hard to compare absolute IPDs among studies and measurements. Secondly it makes PS indistinguishable from other related constructs such as extra-personal monitored space far from the individual, or peri-personal space, a near-body space with protective and connective functions [17]. Thirdly, if PS size ranges spontaneously at about 65 cm, as Hall [3] proposed, then the applicability of proxemics for purposes of human factors, such as interior design or safety zones in public places [18], or clinical diagnostics [19, 20] would be quite limited. The first issue addresses a problem of reliability, the latter two problems concern construct validity of preferred IPD as a measure of PS size.

Hayduk [5] has attempted to address the issue of reliability by comparing test-retest correlations in a multitude of studies. For IPD, as measured by the stop-distance paradigm, this reliability was particularly high, at about .81. However, this concerns only the stability of rank-orders and differences within the sample, which cannot address why measurements seem to differ on an absolute scale. With that in mind, the *first aim* of our study was to quantify absolute reliability, the degree to which the measurements deviate on an absolute scale [21, 22]

### IPD and discomfort

The latter part of Hayduk's defintion of PS, stating that intrusion of PS elicits arousal and discomfort, has not been sufficiently investigated. Studies in this domain have merely sampled a few points of the IPD continuum [23–25] and did not compare specific functions, which would be needed to gain insight into how discomfort rises as a person feels intruded upon. For example, Hayduk [24] let subjects estimate three distinct points of uncomfortableness to illustrate an intrusion-discomfort relationship. Subjects walked towards a confederate and told the experimenter when they felt slightly, moderately, or very uncomfortable. Distances for those three points showed an increase of uncomfortableness associated with intrusion into PS. This is in line with equilibrium-theory, which suggests that preferred IPD can be seen as an equilibrium of approach and avoidance forces regulating the level of intimacy [26]. Any deviation from the equilibrium-point should increase discomfort. This suggests that outside of PS, discomfort should increase again. Note that the notion of discomfort when moving away from a person does presuppose an action goal to interact with this person. The social situation should be standardized such that the action goal can be considered constant.

For lack of a good term, we refer to a position too far from the equilibrium point as extrusion. We hypothesize that intrusion and extrusion will both reduce the subjective comfort of the subject. The question is whether discomfort rises asymmetrically as one moves away from the comfort spot or equilibrium-point toward intrusion or toward extrusion. As there is more room for extrusion, we would expect the gradient to be shallower in extrusion cases compared to intrusion.

To our knowledge, there are no published studies that provide a clear answer to this question, although several attempts have been made to measure comfort. For example, Thompson et al. [27] manipulated IPD between people interacting in video scenes and asked subjects to judge comfort and appropriateness of the distances depicted. They found large (300 cm) and short distances (between 0 and 180 cm) to be less preferable, as compared to intermediate distances (180–240 cm), which were rated as most pleasant. Their findings suggest that there is some tolerance space around the preferred distance [28, 29]. This tolerance for violations could possibly explain why IPD differs in active and passive stop-distance tasks. In Iachini et al. [30], passive approaches by the confederate, whom the subject signaled to stop, resulted in larger distances as compared to approaches where the subject walked towards the confederate. Alternatively, the differences could be due to poor reliability of the IPD measure. Thus, the active approach used in a stop-distance task should be supplemented with a passive approach, and it should be replicated. Against this backdrop, the *second aim* of our study was to examine the function of IPD in relation to discomfort using the stop-distance task in both active and passive approaches.

## Method

### Sample

We recruited 24 subjects at the University of Mainz aged from 18 to 28 years (*M* = 21.66, *SD* = 6.92, 6 male), with an average body height of 170.96 cm (*SD* = 7.25 cm). Prior to testing, they gave written consent in accordance with the declaration of Helsinki and filled out a demographic questionnaire. Prior to the study, the Institutional Review Board (IRB) of the Institute of Psychology at the University of Mainz had informed us that in accordance with the department's ethics guidelines no explicit ethics vote of the IRB was necessary for our study, because we designed the experiments to test healthy adult volunteers, to present only harmless visual stimuli, to rule out physical or psychological stress, and to refrain from measuring physiological parameters. We did not intend to collect sensitive data like personality or clinical scales, or to provide misleading or wrong information to participants. All subjects reported their acquaintance with the confederate (good friend–mere acquaintance—stranger). All participants rated the confederates to be strangers. They had normal or corrected-to-normal visual acuity (Snellen fraction 1.0 or larger) as measured by the Freiburg Acuity Test [31] and they received partial course credit for participation.

### Design and stimuli

**Fig 1:**
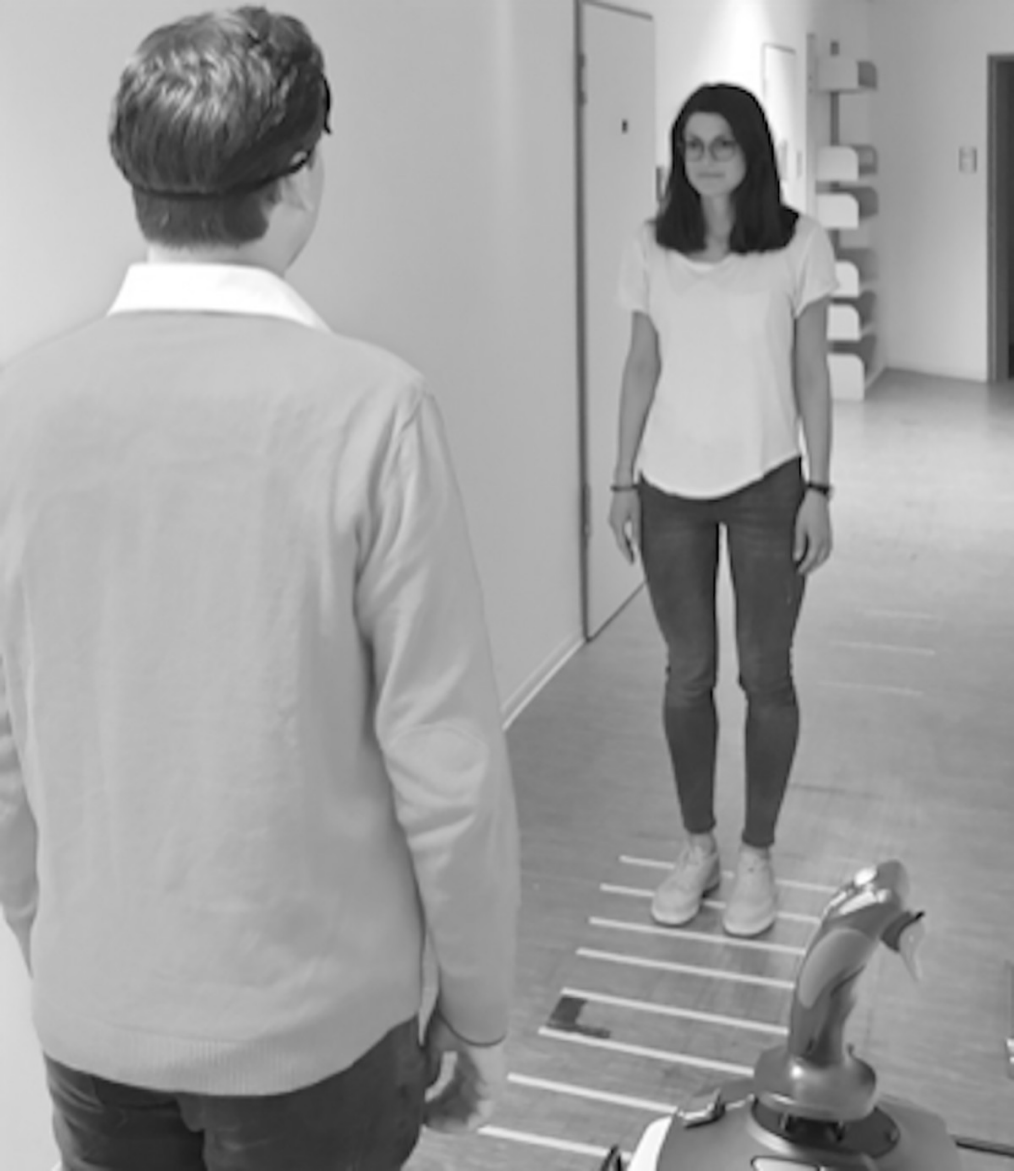
Schematic representation of the confederate standing in the rear and the subject in the front with blindfold (Block 1). Tape on the floor marked the 15 IPDs.

Subjects were placed at 15 frontal IPDs to a confederate varying from 40 cm to 250 cm in steps of 15 cm, which corresponds to the mean minimum and maximum distance for conversation obtained by Williams [11]. These distances were marked–but not labelled–with tape on the floor. On a given trial, both subject and confederate were positioned on a random pairing of these marks aligned to their body-center. The body-center was estimated to be the middle of the foot, marked by dots on the shoes. Subjects as well as the confederate were instructed to look straight at each other’s face throughout the whole experiment. The two confederates taking part in this study were both young females. One of the confederates was 165 cm in height and had blond hair, the other was 167 cm tall and had brown hair. The two confederates took turns between sessions in order to counteract potential confounding variables, i. e. fatigue, poor concentration, etc. Both confederates wore a white shirt and blue jeans, see Fig 1. The individuals in this Figure have given written informed consent (as outlined in PLOS consent form) to publish this photograph.

### Procedure

For all testing blocks, we standardized the social situation to minimize situational effects on IPD [3]. Subjects had to imagine a scenario in which they were in an open space in an unfamiliar city asking a stranger for directions. Subjects were placed at 15 different IPDs in a fixed-distance task and were asked to rate subjective discomfort verbally on a rating scale ranging from -100 (maximum discomfort, too close) to 0 (ideal distance) to +100 (maximum discomfort, too far). In Block 1, the subject was directed by the experimenter and the confederate remained stationary. In Block 2, the subject remained stationary and the confederate moved to the respective positions between trials. Subjects were blindfolded during the positioning. After the positioning, the blindfold was lifted and he/she rated subjective discomfort.

Block 3 followed the procedure of Block 2, but subjects rated discomfort by positioning a joystick. This was done to control for social desirability, the confederate was unable to see the exact tilt of the joystick. Subjects were instructed to tilt the joystick away from themselves as a function of experienced discomfort when IPD was deemed too close, or to tilt the joystick towards themselves when the distance was not close enough. All possible orders of Blocks 1, 2 and 3 were used and counterbalanced between subjects. Within each block, the order of distances was randomized.

Next, subjects completed two repetitions of an active and a passive stop-distance task to estimate the preferred IPD. In the active stop-distance task, the subject approached the confederate until comfortable IPD had been reached. In the passive stop-distance task, the subject was slowly approached by the confederate until the subject signaled the confederate to stop. Subjects were allowed to fine-tune this distance by instructing the confederate to adjust forward or backward. Preferred IPD was measured via a tape measure on the floor and recorded as the distance between the subject’s and the confederate’s body center. Order of the passive and active stop-distance task was counterbalanced within the sample. Subjects were tested in individual sessions of approximately 60 minutes. No time constraints were imposed in any of the trials [24]. After the procedure, the subjects were thanked and debriefed. We report all measures and scale manipulations in this study. We did not exclude any of the experimental trials from data analysis and sample size was not increased after data analysis.

### Statistical analysis

To enable the examination of the Null-hypothesis, we have opted for a Bayesian approach to data analysis. The Bayes Factor (BF) is used for statistical inference and is computed using the BayesFactor-package [32, 33] in R [34]. Here, the BF quantifies the relative likelihood of the Null-model as compared to the alternative-model given the observed data. We either provide the likelihood for the Null-model relative to the alternative model (BF_01_) or the reverse fraction (BF_10_). Note that we have compared different weakly informative priors in a prior-sensitivity analysis. The choice of priors did not influence statistical inference in this study as the data obtained clearly overwhelmed the priors when computing the Bayes Factors. Thus, we stuck with the default priors of the BayesFactor-package in *t*-tests, regressions and analyses of variance. We report median estimates of parameters with high density intervals at 95% from the posterior distribution. To model the relation of IPD and discomfort, we calculated a Bayesian linear mixed model (BLMM) using brms [35], a wrapper for the STAN-sampler [36] for R [34]. We applied normally distributed priors (*M* = 0, *SD* = 1) on all beta-coefficients, with Cholesky priors on the residual correlation (η = 1) and a t-distributed prior to allow for thicker tails (*df* = 3, *M* = 0, *SD* = 10) on the centered intercept, the variance parameters and sigma. These priors are only very weakly informative and mostly help in the regularization of the posterior distributions. We computed 4 Hamilton-Monte-Carlo chains with 10000 iterations each and 20% warm-up samples. Trace plots of the Markov-chain-Monte-Carlo permutations were inspected for divergent transitions. All Rubin-Gelman statistics were well below 1.1. The experimental data and the R code can be found in the Supplementary Material S1 Data and S1 Code. The files provided comprise the minimal underlying data that an independent researcher would need in order to replicate all of our results, conclusions, figures and summary statistics. The files do not contain any personally identifying information.

## Results

### Reliability of the stop-distance tasks

First, we will consider relative reliability, stability of rank order and differences within the sample, and second, we will consider absolute reliability, which refers to the absolute deviation of sequential measurements.

**Fig 2:**
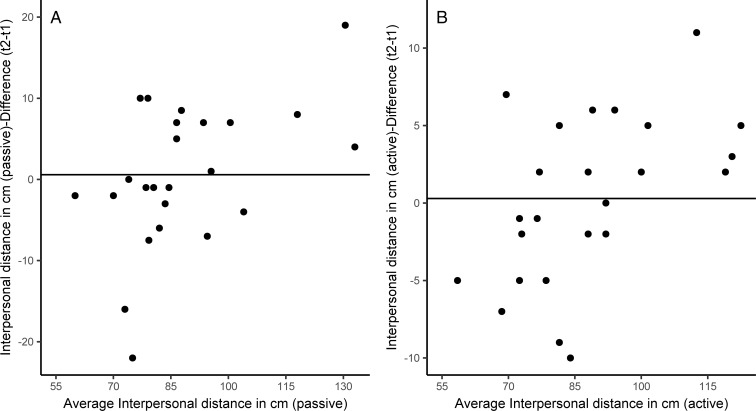
Difference of IPD from both test and retest as a function of averaged IPD of both tests for every participant (Panel A: active stop distance task; Panel B: passive stop distance task) with mean difference (black line).

Test-retest reliability and thus the relative reliability, as measured across the two repetitions, was high in both the passive, $\tilde{r}$ = .85 [.70; .93], BF_10_ > 100, and active condition, $\tilde{r}$ = .94 [.87; .97], BF_10_ > 100, of the stop-distance tasks. Concerning absolute reliability, Fig 2 shows that the individual mean IPD of both trials and the differences between repetitions 1 and 2 were largely independent in both tasks, see panels A and B. This indicates that the variation within subjects between repetitions was unrelated to the variation between subjects, and thus within-subjects variation was probably unsystematic.

Spontaneous variation was rather small in all tasks, in the range of +/- 10–15 cm, and unrelated to the size of PS. Thus, a change in IPD in this range should be detectable as a violation of PS. To investigate a potential difference in absolute reliability between tasks, we computed a Bayesian two-way repeated measures analysis of variance (BrmANOVA) with the factors approach (active vs. passive) and test (test vs. retest). However, a null-model was more likely to be true given the data than were models with main effects, BF_01_ > 4.1 or interaction effects, BF_01_ > 18.57. Thus, differences in measurements of test and retest varied unsystematically and not as a function of active and passive approaches. Contrary to Iachini et al. [30], active and passive approaches did not produce any differences in preferred IPD at all.

### Construct validity

**Fig 3:**
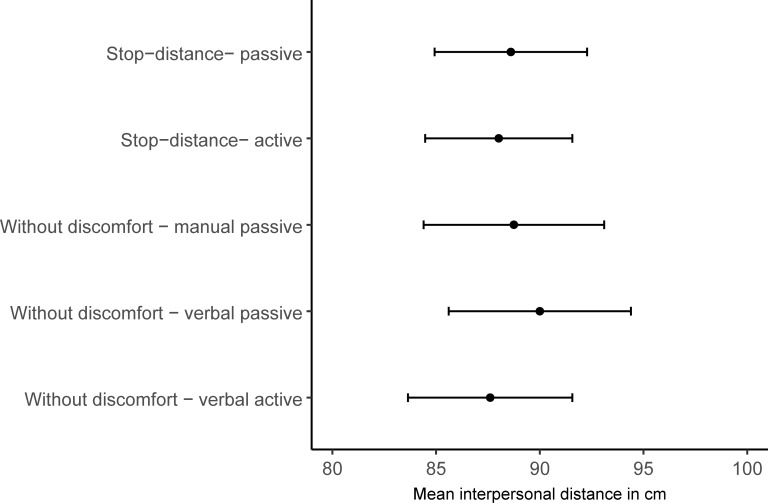
Mean preferred interpersonal distance as a function of Block. Error bars denote +/- one standard error of the mean.

The stop-distance task seems to reliably measure the outlines of personal space. But what point does the stop-distance task sample from the continuum of IPD and discomfort? We compared the mean shortest distances that did not elicit any discomfort (Block 1, 2, 3) to the mean IPDs of the stop distance trials (Block 4–5) for each individual, see Fig 3. A one-way BrmANOVA again favored the null-model against a model that assumed differences in IPD across Blocks, BF_01_ > 27.60.

**Fig 4:**
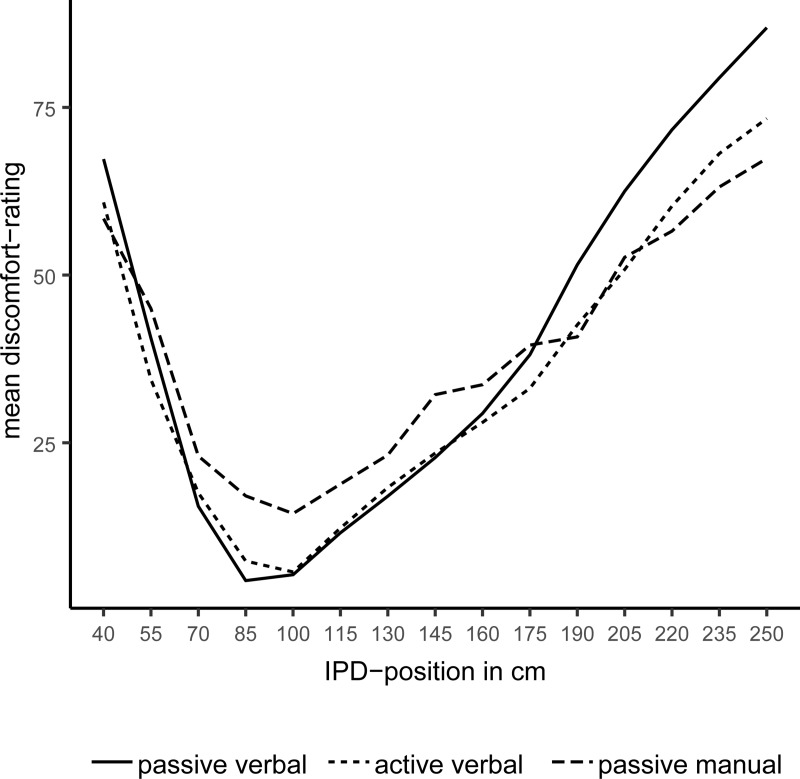
Mean discomfort ratings as a function of interpersonal distance (IPD) separately for each block, aggregated across subjects. Lines denote the different methods of assessment for discomfort applied in the three blocks.

The mean IPD in the stop-distance measures (aggregated across active and passive approaches; *M* = 88.31, *SD* = 17.39) and the mean shortest distance without discomfort, (aggregated across the three blocks; *M* = 88.33, *SD* = 16.08) did not differ significantly, BF_01_ = 4.63, $\tilde{\delta }$ = 0.02[-0.39; 0.35]. Thus, the shortest distance in the discomfort-function does roughly correspond to the edges of PS as measured by the stop-distance task, $\tilde{r}$_spearman_ = .55 [.21; .77], BF_10_ = 64.31. This alone does not fully qualify the absence of a tolerance for violations of PS beyond the minimum point of discomfort. To fully dismiss a tolerance in violations of PS, we visualized the standardized discomfort ratings for every block, and averaged them across subjects for every distance presented, see Fig 4.

**Fig 5:**
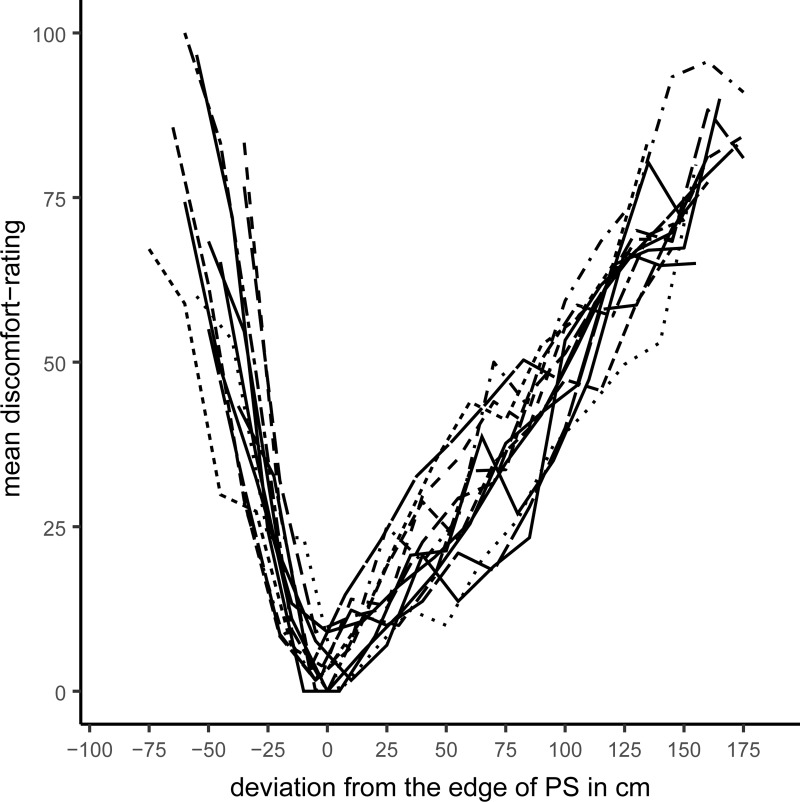
Individual discomfort ratings as a function of deviation from the edge of PS, for each of the 24 subjects, aggregated across blocks.

This plot shows active verbal ratings, passive verbal ratings, and joystick-based ratings of discomfort as a function of IPD. Descriptively, the shape of the discomfort-function aggregated across subjects does not vary across Blocks, indicating that the ratings were unaffected by our manipulation of response modality. Short and large IPDs tended to increase discomfort. A valley in the function occurred at distances around 85 to a 100 cm, which indicates a certain tolerance for violations of PS. However, we entertain that the valley with least discomfort in Fig 4 resembles the between-subjects variance, see the error bars in Fig 3. It follows that the apparent U-shape could have been produced by the averaging of varying individual V-shaped data. To investigate this potential effect of aggregation across subjects, we inspected the 24 individual curves of all subjects in every Block, and centered these functions on the edge of the individual PS, that is on the shortest individual mean IPD (averaged across the three Blocks) that did not elicit any discomfort (see Fig 4). Inspecting the individual centralized functions in Fig 5, it becomes clear, that the U-shaped pattern observed in Fig 4 appears to be an artifact caused by the aggregation across subjects.

We modeled the relation of distance and discomfort separately for intrusion and extrusion. We also compared a linear vs. an exponential relationship between distance and discomfort. In the BLMM, we estimated a varying intercept for every subject to account for the repeated-measures structure of the data. Discomfort was predicted from PS intrusion/extrusion, the deviation from the edges of PS in cm. A linear fit seems to be more than 100 times more likely (BF_lin/exp_ > 100) than an exponential fit, for both intrusion and extrusion of personal space, given the empirical data and our priors. For intrusion, $\tilde{R}$^*2*^ = 66.60% [61.57; 70.33], there was a steep linear increase in discomfort $\tilde{b}$ = 1.35 [1.22; 1.48]. A weaker link of discomfort and distance was found in the extrusion model, $\tilde{R}$^*2*^ = 75.64% [72.90; 76.7], $\tilde{b}$ = 0.46 [0.44; 0.48]. To put it more precisely, for every centimeter of intrusion discomfort rose at about 1.35 points. For every centimeter of extrusion, discomfort increased at about 0.46 points.

Next, we analyzed discomfort in response to intrusion and extrusion in a joint model, which let us directly compare the steepness of slopes for intrusion and extrusion. In contrast to the previous models, we specified deviation from the edges of PS, intrusion vs. extrusion (coded as -0.5; +0,5), and their interaction as population-level effect. In addition to the varying intercept for every subject, we added varying slopes for all population-level effects. Furthermore, we z-standardized distance and discomfort, which let us compare the functions for intrusion and extrusion. In total, this model explained $\tilde{R}$^*2*^ = 74.60% [72.96; 75.90] _95% HDI_ of the variance in the data. We now take a closer look at the population-level effects. Deviation from the edges of PS did increase discomfort, $\tilde{b}$ = 1.64 [1.50; 1.78], and discomfort was slightly larger in intrusion trials as compared to extrusion trials, $\tilde{b}$ = 2.09 [1.73; 2.45]. Most importantly, the slope was $\tilde{b}$ = 1.66 [1.39, 1.95] times steeper for intrusions than for extrusions of PS. Thus, we can conclude that PS is anisotropic with respect to intrusions and extrusions.

## Discussion

Unlike previously thought, the response to violations of PS is rather immediate. That is, our data do not support the notion of a tolerance zone around the preferred IPD where intrusion or extrusion is acceptable in the sense that it leaves comfort ratings unaffected. Most importantly, preferred IPD in the stop-distance tasks corresponds to the shortest distance without discomfort in the rating-task. Spontaneous variations in IPD occur in the range of 10–15 cm and seem to be unrelated to the mean IPD (see Fig 2). Thus, the stop-distance task is rather reliable and seems to produce a valid approximation of the borders of PS. The tolerance for violations of PS previously observed in other studies [27], may merely be an artifact of aggregation across subjects, which may mislead into suspecting a larger acceptable range or even a U-shaped function of IPD and discomfort. Note, however, that we cannot rule out a tolerance for violations of PS smaller than 15 cm as we sampled distances in steps of this size. Within this range, spontaneous variations in preferred IPD may occur. Furthermore, we merely sampled distances from 40 cm to 250 cm and found a linear increase of discomfort with deviation from PS, this might not hold for extrusions of more than 200 cm.

An intrusion into PS of 15 cm or more beyond the comfort point leads to an immediate steep increase in discomfort. Movement in the opposite direction away from the other person leads to a likewise immediate but shallower increase in discomfort. Thus, the response to intrusion and extrusion is anisotropic. Equal distances from the border of PS produce unequal increases in discomfort. Intrusion has a steeper gradient than extrusion. Let us substantiate this idea with the example of a conversation between person A and person B. If person A reduces IPD toward person B, the probability for a corrective step by B away from person A should increase immediately. If, on the other hand, person A enlarges IPD toward person B, the probability for a corrective step by B toward person A should likewise increase, but with a lower probability than in the first scenario. Because extrusion of PS does not produce as much discomfort as does intrusion of PS.

In other words, we predict a hysteresis effect in the following sense. We have always used an approach scenario, that is in active and passive approach in which the initial IPD was larger than the ideal IPD. If one were to start the stop-distance task once from a position well within the intrusion zone and once from a position within the extrusion zone, we would expect slightly larger preferred IPD in the latter case.

Note that the anisotropy of PS holds with respect to intrusion/extrusion but not with respect to active/passive approach. Contrary to Iachini et al. [30], we could not find any differences in active and passive approaches. This could be due to the habituation of subjects to our stimulus. Whereas our confederate completed all experimental trials, their stimuli randomly changed throughout the experiment. Thus, effects of perceived dominance or potential fear of the approaching target, which may be particularly salient in active approach, may have already faded in our experiment. This might also explain the comparatively large (i. e. more conservative) judgments of preferred IPD in their experiments.

Following the examination of the relation of IPD and discomfort, we can qualify some proxemic theories. Preferred IPD has been seen as an equilibrium of approach and avoidance forces regulating the level of optimal stimulation [37, 38]. Accordingly, the deviation of IPD from the point of equilibrium has been taken to produce equal discomfort on the intrusion and extrusion side, which was not supported by our data. Sundstrom et al. [39] as well as Thompson et al. [28] suggested a U-shaped relation of IPD and discomfort. They also proposed some degree of sluggishness or tolerance for violations of PS [40]. Short distances from the equilibrium-point should affect discomfort to a lesser degree than large distances. Again, we could not find any support for this prediction in our data.

We like to entertain a different view on PS. We suggest that PS behaves like a dynamically self-constructed space. This space surrounds the person and can be characterized in terms of its shape [7], elasticity [12], and density with repelling and attracting forces. Kurt Lewin [41] has attempted to formalize the notion of psychological spaces in his field theory, wherein human behavior within the environment is characterized by vectors of approach or avoidance forces that tend toward a state of equilibrium [26]. These vectors are tied to individual perception and constitute non-Euclidian psychological distances that partition the environment into different psychological fields or spaces. Thinking of PS in field-theoretical terms, we may be able to quantify the principles of maintaining and constructing a PS. In the present study, our measure of IPD can be interpreted as the equilibrium-point where approach and avoidance forces on the individual are balanced. The force gradient as one moves away from this equilibrium-point is roughly linear–at least within the distances we have sampled–and it is anisotropic. It is steeper on the intrusion side of the equilibrium point than it is on the extrusion side. This might be because avoidance tendencies tied to intrusions of PS, and approach tendencies related to extrusions, are weighted differently in producing discomfort. Furthermore, approach and avoidance forces are fueled by a large set of determinants in social interactions, such as the urgency of the communication, the level of intimacy, fear, arousal, etc., for a review see Hayduk [5, 10]. How exactly approach and avoidance relate to IPD and discomfort is beyond the scope of this study, but future investigations may embark upon this problem from a field-theoretical perspective.

Within this field-theoretical framework, we can generate qualified hypotheses as to the effects of a given person variable or a given environment variable. For instance, it might make sense to hypothesize on the basis of what is known about psychopathy, that the equilibrium-point is unaltered in psychopathic subjects but the gradient is much shallower on the intrusion side than it is in less psychopathic subjects when confronted with social threat [12]. In contrast, external factors, such as the crowdedness of the space, could merely move the equilibrium-point without affecting the steepness of the gradient. For example, in a crowded market place the equilibrium-point should be closer to the person. Future studies should refine this field-theoretical model and test the novel predictions with regard to discomfort and IPD, which it allows to generate.

## Supporting information

S1 Data Data of the experiment. (ZIP)

S1 Code R-syntax for data analysis. (R)
